# Point-of-care Cranial Ultrasound in a Hemicraniectomy Patient

**DOI:** 10.5811/cpcem.2018.7.39379

**Published:** 2018-09-10

**Authors:** Aarti Sarwal, Natalie M. Elder

**Affiliations:** * Wake Forest School of Medicine, Department of Neurology, Winston-Salem, North Carolina; † Wake Forest School of Medicine, Department of Emergency Medicine, Winston-Salem, North Carolina

## CASE PRESENTATION

**Image 1:**
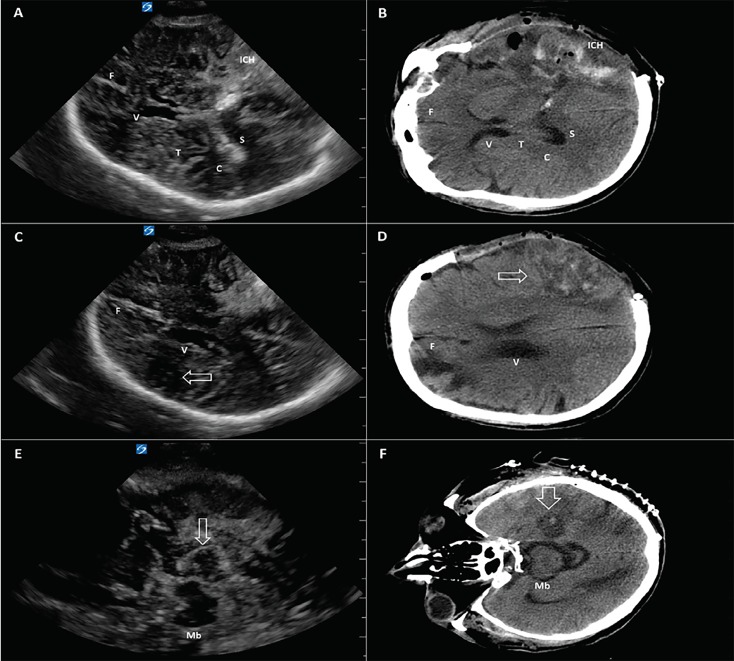
Cranial ultrasound through the left transtemporal window (left column, panels A, C, E), with corresponding cross-sectional anatomy on a non-contrast computed tomography of brain (right column, panels B, D, F) in a 74-year-old male with a left hemicraniectomy. Intracranial hemorrhage (ICH) and hypodense ischemic areas are indicated by white arrows. *F*, falx cerebri; *V*, lateral ventricles; *Mb*, midbrain; *C*, choroid plexus; *S*, splenium of corpus collosum; *T*, thalami.

**Image 2:**
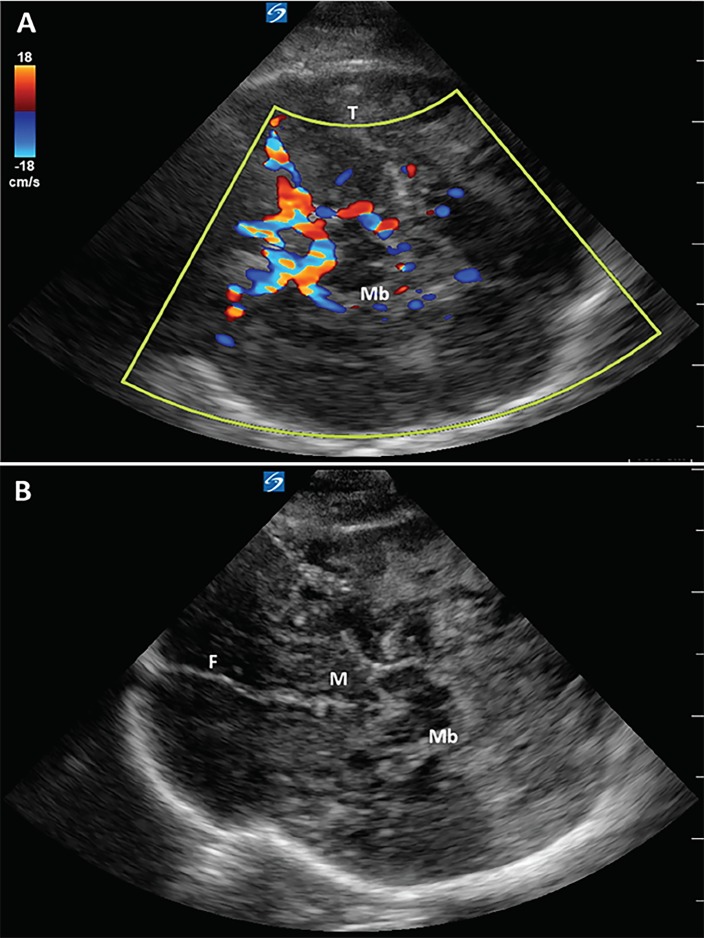
Cranial ultrasound with Doppler (panel A) and without Doppler (panel B) through the left transtemporal window in a 74-year-old male with a left hemicraniectomy with additional anatomical details. *T*, thalami; *Mb*, midbrain; *F*, falx cerebri; *M*, mammillary bodies.

A 74-year-old male presented to the emergency department with right-sided weakness and confusion and was found to have a left parietal intraparenchymal hemorrhage with cerebral edema and left-to-right midline shift on non-contrast computed tomography (CT) of the head. Increase in cerebral edema and expansion of the hematoma caused clinical neurological decline necessitating a left-sided hemicraniectomy with clot evacuation. A cranial ultrasound was performed two days after surgery to assess for progression of cerebral edema and intracranial hemorrhage. A transtemporal approach in axial plane was used to visualize intracranial structures through the craniectomy window (Images 1 and 2). Physiological structures such as the falx cerebri, lateral ventricles, midbrain, mammillary bodies, choroid plexus, splenium of corpus callosum, thalami, and circle of Willis were visualized with incredible anatomical detail. Pathologies such as intracranial hemorrhage, focal ischemic areas, and vasogenic edema, as well as encephalomalacia, were identified with close correlation to the non-contrast head CT. The patient is currently recovering in the neurocritical care unit with supportive care.

## DISCUSSION

Visualization of intracranial structures by ultrasound in adults is limited by the presence of skull, although ultrasound imaging can occur through temporal windows. Point-of-care ultrasound allows for the assessment of midline shift, brainstem, and ventricles, and Doppler allows visualization of cerebral perfusion patterns.^1^,^2^ Patients with a hemicraniectomy have better temporal windows available since a portion of their skull has been removed. In such patients, ultrasound can provide a non-invasive method to serially assess midline shift, intracranial hematomas, and focal ischemia at the bedside.

**CPC-EM Capsule**

What do we already know about this clinical entity?

Point-of-care ultrasound (POCUS) is widely used in the emergency departments (ED) as a tool to visualize anatomy without exposing patients to potentially harmful radiation.

What is the major impact of the image(s)?

The ability to view anatomical structures and pathology of patients with hemicraniectomies using POCUS may decrease radiation from repeat computed tomography.

How might this improve emergency medicine practice?

POCUS scans on patients with hemicraniectomies will allow for faster assessment of structures, pathology and cerebral perfusion in the ED.

Cranial ultrasound has potential applications in point-of-care assessment of intracranial pathology in neurocritical care patients. This application has promising use in directing therapy in patients who are otherwise unstable for transport and may provide a noninvasive, radiation-free diagnostic tool for serial neuroimaging.

Documented patient informed consent and/or Institutional Review Board approval has been obtained and filed for publication of this case report.

## References

[1] D’Andrea A, Conte M, Scarafile R (2016). Transcranial Doppler ultrasound: physical principles and principal applications in neurocritical care unit. J Cardiovasc Echogr.

[2] Kalanuria A, Nyquist PA, Armonda RA (2013). Use of transcranical Doppler (TCD) ultrasound in the neurocritical care unit. Neurosurg Clin N Am.

